# Less Sugar and More Whole Grains in Infant Cereals: A Sensory Acceptability Experiment With Infants and Their Parents

**DOI:** 10.3389/fnut.2022.855004

**Published:** 2022-05-13

**Authors:** Luisma Sanchez-Siles, Sergio Román, Juan F. Haro-Vicente, Maria Jose Bernal, Michelle Klerks, Gaspar Ros, Ángel Gil

**Affiliations:** ^1^Research and Nutrition, Hero Group, Murcia, Spain; ^2^Institute for Research and Nutrition, Hero Group, Lenzburg, Switzerland; ^3^Department of Marketing, Facultad de Economía y Empresa, University of Murcia, Murcia, Spain; ^4^Department of Food Technology, Nutrition and Bromatology, Faculty of Veterinary, University of Murcia, Murcia, Spain; ^5^Center of Biomedical Research, Institute of Nutrition and Food Technology “José Mataix”, University of Granada, Granada, Spain; ^6^Department of Biochemistry and Molecular Biology II, School of Pharmacy, University of Granada, Granada, Spain; ^7^ibs. GRANADA, Instituto de Investigación Biosanitaria, Complejo Hospitalario Universitario de Granada, Granada, Spain; ^8^CIBEROBN (CIBER Physiopathology of Obesity and Nutrition), Instituto de Salud Carlos III, Madrid, Spain

**Keywords:** sensory acceptability, sugar reduction, cereals, complementary feeding, whole grains, public health

## Abstract

There is an urgent need to reduce sugar intake in early childhood. Commercial infant cereals are among the first solid foods introduced to infants at the beginning of the complementary feeding period in most countries. The aim of this study was to examine infants’ overall acceptability of low-sugar complementary cereals. To do so, a between-subjects experimental study with 165 parents and their infants aged 6–24 months was conducted where one group tested a high-sugar refined cereal (21 g/100 g), and the other a low-sugar cereal (<1 g/100 g) with 50% of whole grain, which represented a 95.2% decrease in sugar content. We found no significant differences between the two groups in terms of infants’ overall acceptability (infant’s reaction, estimated intake and relative intake). Importantly, infants’ reactions to high- and low-sugar cereals were not influenced by the time that infants had been consuming sweet cereals (15–25% sugar) before the experiment took place. In addition, parent’s overall liking and sensory evaluation (sweetness, color, taste, texture, and aroma) was positive and very similar in both groups. Overall, our findings show that it is feasible to reduce sugar content in infant cereals without sacrificing its sensory acceptability by infants and their parents. This represents a good opportunity for the infant food industry to adhere to current healthy and sustainable demands of lowering the sugar intake leading to important benefits in infants’ health, without compromising competitiveness in the market.

## Introduction

Excessive sugar consumption has been long associated with many diseases such as obesity, diabetes, high blood pressure, dental caries and cholesterol (1, 2). Accordingly, regulatory bodies, public health authorities, and professional organizations have been calling for a reduction in the sugar content of processed foods (3–5). Such reduction has straight-forward health benefits, but represents major challenges for the food processing industry (6–9). In particular, there might be technical difficulties as sugar’s role in foods is not restricted to providing sweetness only; it has other functions such as providing texture, color, and stability (8). For instance, the enzymatic hydrolysis of infant cereals (which produces free sugars) was traditionally carried out to stabilize their viscosity after preparation and for the physiological aim to increase the starch digestibility (10). Hence, the development of technical solutions to reformulate foods requires know-how and resources which may increase the product’s costs. Factors such as consumer education and legislation also play important roles in the success of reformulation strategies of sugar-reduced foods (9). Still, consumer acceptance becomes a fundamental challenge for food manufacturers. That is to say, consumers might dislike the taste of sugar-reduced foods, and thus may not purchase the reformulated/healthier version. For instance, findings from Markey et al. p. 138, who examined consumers’ acceptability and purchase intention of several sugar-reformulated foods and drinks, evidenced that: “significant improvements in the sensory qualities of some sugar-reduced products are required before their acceptance [by consumers]” (11), while Hutchings et al. p. 2,287 review concluded that: “substantial reduction of sugar in processed foods without compromising sensory properties may be an impossible dream” (12).

Only recently, scholars have paid attention to analyzing consumers’ sensory and hedonic responses to sugar-reduced foods (a summary of relevant studies is provided in Table 1). These studies provide very interesting insights, but their findings are not always consistent. In particular, while lowering sugar content did not affect sensory acceptance or liking in some studies (13–17), others reported mixed findings (18–25). For example, Oliveira et al. found that sensory perception was negatively affected for sugar content reductions higher than 10% and overall liking significantly decreased when reductions were higher than 20%, but not in lower reductions (23). Oliveira et al. p. 8 evidenced that sugar reductions up to 25% were accepted by consumers if 0.2% of flavor were added to the yogurts and still the modified yogurts were perceived as less sweet, and some subjects detected “unpleasant tastes” (26). In fact, other studies have even shown that sensory perceptions were negatively affected, and overall liking significantly reduced (11, 27–29).

**TABLE 1:** Summary of studies evaluating consumers’ sensory and hedonic reactions to sugar-reduced foods.

References	Product	Reduction% (additional changes)	Sample	Main results
Chollet et al. (19)	Flavored yogurt	30; 50%	197 and 256 consumers (52% women; from 15 to > 60 years old) Switzerland	Consumers accepted flavored yogurts with 7% of added sugar, as compared to 10%; but yogurts with 5% of added sugar were not accepted.
Biguzzi et al. (27)	Biscuits	9.8–29%	79 consumers (mostly women; mean age of 42.5 years) France	It was more acceptable to reduce the fat than the sugar content in biscuits from a sensory point of view.
Biguzzi et al. (18)	Biscuits	9–28%	106 consumers (mostly women; mean age of 38.46 years) France	Consumers’ liking of biscuits only improved for 9 and 16% sugar-reduced variants.
Klerks et al. (17)	Fruit yogurt pouches	23–30%	150 parent-toddler (1–4 years) dyads in Spain	A reduction of sugar content up to 30% along with a reduction in the number of processed ingredients is acceptable by toddlers and their parents.
Markey et al. (11)	Baked beans, jam, chocolate, cola and fruit juice	32–100%	116 consumers (52% female; mean age of 33 years) UK	A high proportion of consumers prefer conventional products over sugar-reduced products across a wide range of product types.
Oliveira et al. (22)	Probiotic chocolate-flavored milk	20; 40; 60%	100 consumers (65% female; 15–43 years old) Uruguay	A reduction in added sugar of 20% led to changes in sweetness intensity. However, consumers’ liking was not largely influenced by sugar reduction up to 40%.
Pineli et al. (13)	Orange nectar	15%	231 men and women (18–34 years old) Brazil	Lowering sugar from 10 to 8.5% did not affect acceptance or sensory attributes.
Wise et al. (25)	Low sugar diet over 5 months	40%	29 men and women (21–54 years old) United States	Reduced dietary intake of simple sugars alters perceived sweet taste intensity but not perceived pleasantness.
Romagny et al. (14)	Muffins	25%	144 adult consumers (58% female; 20–70 years old) France	No significant differences between the non-reformulated version and reformulated version was observed for the pleasantness rating.
Lima et al. (21)	Grape nectar	26.3% in adults 45.4% in children	105 children (62% female; 6–12 years old) and 100 adults (67% female; 18–65 years old) Brazil	Children’s overall liking scores significantly decreased with added sugar reduction. However, significant differences from the control nectar were only found when sugar reduction reached 45.4%. Adult’s liking was not influenced by sugar reduction. Children were less able to detect changes in the sensory characteristics of sugar-reduced samples than adults, but evidenced higher hedonic sensitivity to sugar reduction.
Oliveira et al. (23)	Passion fruit, orange and pomegranate nectar	2.56–20%	300 adult consumers (59% female; 18–60 years old) Brazil	An increase in the frequency of use of the terms barely sweet, watery and acid taste was found in sugar reductions higher than 10%. No significant differences in overall liking were detected for fruit nectars with 20% sugar reduction. Hedonic reactions were consumer and product dependent.
Oliveira et al. (29)	Orange/passion fruit nectar	20; 40%	206 adult consumers (70% female; 18–66 years old) Brazil	Overall liking scores were significantly lower in the sugar-reduced samples (20 and 40% reduction) compared to the control sample.
Lima et al. (28)	Grape nectar	57%	147 children (46% female; 6–12 years old) Brazil	Reducing the added sugar content led to a decrease in sweetness and an increase in acidity and watery, which resulted in a decrease in overall liking.
Sanchez-Siles et al. (15)	Infant cereals	50% (an increase of 50% of whole grain)	46 infants and their parents (mean age of 5.2 months) Spain	Lowering sugar from 24 to 12 g did not affect the sensory acceptability of infants and their parents.
Velazquez et al.(24)	Vanilla milk desserts	41.6%	112 children (8–12 years old) Uruguay	The reduction of added sugar had no significant effects on children’s hedonic reactions and only minor consequences on sensory perception.
de Souza et al. (20)	Strawberry yogurt	14;40%	121 adult consumers (53% female; mean age of 23.8 years) Brazil	Reductions up to 14% of sugar were accepted by consumers, but 40% were not.
Mahato et al. (16)	Chocolate flavored milk	50% (5–100 ppm stevia sweetener and 50–100 ppm monk fruit extract were added)	107 adult consumers (64% female; 20–65 years old) Australia	Subjects accepted sugar reduction when the concentrations of added stevia sweetener and monk fruit extract were 56.27 ppm and 81.90 ppm, respectively.
Oliveira et al. (26)	Strawberry and vanilla yogurt	25; 50%; (0.1 and 0.2% of flavor were added)	91 adult consumers (55% female; 18–50 years old) Brazil	Sugar reductions up to 25% were accepted by consumers if 0.2% of flavor were added; the remaining combinations were not accepted.

Most likely, these varied reactions from consumers stem from the differences in terms of the (sugar-reduced) products tested (biscuits, fruit nectars and juices, chocolate, cola soft drinks, muffins, chocolate-flavored milk, milk desserts, baked beans and infant cereals), percentage of reduction in sugar (ranging from 2.56 to 100%) and consumers’ age and gender (adults, children, and infants) as evidenced in prior research (23, 29). Two key implications can be derived from this increasingly relevant stream of research: sugar reduction strategies are far from easy to be implemented and more research is needed in this regard, particularly in under-researched population segments such as children and infants (30).

All previous studies on consumers’ acceptability of sugar-reduced foods have been carried out mostly with adults and to a minor extent with children as shown in Table 1. One notable exception is Sanchez-Siles et al. who evaluated the sensory acceptability of infant cereals with a sugar reduction of 50% (from 24 g/100 g to 12 g/100 g) (15). Their preliminary findings on 46 infants from one Spanish city suggest that sugar reductions are feasible. Similar to that study, our interest in the present work is on infants (and their parents). Notably, food preferences are formed in infancy and shape later food preferences (31, 32). Exposure to sweet products early in life can promote a preference for sweet foods (33) as well as poor eating habits in childhood (34) which could lead to the development of many diseases as argued earlier. There is a need, therefore, to limit sugar intake, particularly in early childhood in order to promote lasting healthy eating habits (35, 36). Also, exposure to less sweet foods in infancy could reduce food neophobia/avoidance of bitter taste later in life (37, 38).

In the complementary feeding period, infants are exposed to a wider variety of foods which increases the diversity of flavor, taste, and texture exposure they receive (39, 40). Interestingly, exposure to sodium in complementary foods was shown to be associated with a higher acceptance of salty taste in preschoolers (41). Commercial complementary cereals play a major role in infant nutrition as they are among the first solid foods introduced at the beginning of the complementary feeding period in most countries (3, 42). Also, cereals are an excellent source of energy and provide starch, fiber, and proteins as well as vitamins, minerals, and other bioactive compounds (43). This is particularly the case for whole grain, which consists of the endosperm, germ, and bran in the same relative proportions as in the intact kernel (44), rather than refined cereals (10, 45). In fact, the consumption of whole grain in infants and children has been emphasized by several public health institutions and professional organizations, e.g., (46–49). Unfortunately, many infant cereals contain high levels of sugar and low percentages of whole grain as evidenced in previous studies and reports (50–53).

In the light of these considerations and facts, the aim of this study is to examine infants’ overall acceptability and parents’ overall liking and sensory evaluations of a drastic reduction of sugar and addition of whole grain in complementary infant cereals. More specifically, we will compare high-a sugar refined cereal (21 g/100 g) with low-sugar cereal (<1 g/100 g) which has 50% of whole grain. Hopefully, our insights can provide policymakers as well as the food industry with valuable guidelines for the effective implementation of policies and actions to reduce sugar and increase whole grain intake in early childhood.

## Materials and Methods

### Participants

Parents with healthy infants from five Spanish cities (Madrid, Barcelona, Seville, Murcia, and Valencia) were recruited through an independent market research firm and from kindergartens. Eligible subjects consisted of parents who had at least one child aged 6–24 months; had primary responsibility for their child’s feeding and their infants were having complementary cereals before the experiment took place. Eligible infants needed to have a gestational age of 37–42 weeks and a birth weight higher than 2,500 grams. Exclusion criteria for participation included: (1) infants who had food allergies or intolerances, swallowing or digestion problems, or other medical issues that could influence the ability to eat and (2) infants who were consuming low sugar cereals [those which contain no more than 5 g of sugar per 100 g according to EU Regulation 1924/2006 (54)] and consequently were already used to this less sweet flavor of cereals. The final sample consisted of 165 parent-infant dyads.

This study was conducted according to the Declaration of Helsinki guidelines. Ethical approval was obtained from the Research Ethical Committee of the University of Murcia (code: CEI 2116/2018). All participants signed an informed parental consent form for each infant before the inclusion. Parents received a 30€ voucher or a gift for their participation.

### Infant Cereals Samples

Two different commercial infant cereals were studied: a high-sugar (21 g/100 g) infant cereal, in which sugar was produced by starch hydrolysis (high-sugar cereal), and a non-hydrolyzed low-sugar (<1 g/100 g) infant cereal (low-sugar cereal). Ingredient information and nutritional composition of the two infant cereals are described in Table 2. Both infant cereals were manufactured in the same production line, with the same processing conditions, and 2 months before testing to minimize quality differences between the samples and recreate normal consumer consumption. The only formulation differences between the two samples were: (1) the level of sugar produced during hydrolysis, and (2) the content of whole grain (0% whole grain in the high-sugar cereal and 50% whole grain in the low-sugar cereal). Nutritional properties of the reformulated low-sugar cereal were slightly better as a consequence of the addition of whole grain (Table 2). All samples used in this study were packaged into identical foil bags. Each bag was marked with a three-digit randomization code. Both samples were labeled equally and were designed and produced by Hero España S.A.

**TABLE 2:** Nutritional composition of infant cereals per 100 g.

Nutrients (per 100 g)	High-sugar cereals	Low-sugar cereals
Energy (kcal)	380	379
Protein (g)	9.1	12.0
Carbohydrates (g)	78	75
Sugars (g)	21	1.0
Fat (g)	2.3	2.2
Fiber (g)	5.2	6.4
Calcium (mg)	160	160
Iron (mg)	6.0	7.5
Zinc (mg)	0.6	1.0
Vitamin A (μg)	375	375
Vitamin D (μg)	10	10
Vitamin E (α-TE mg)	2.8	2.8
Vitamin C (mg)	30	30
Vitamin B1 (mg)	0.5	0.5
Niacin (mg)	8.5	8.5
Vitamin B6 (mg)	0.3	0.3
Folic acid (μg)	70	70
Ingredients	Hydrolyzed cereal flours (wheat, corn, rice, oat, barley, rye, sorghum, and millet), minerals, natural flavor and vitamins	Non-hydrolyzed cereal flour (wheat, whole grain wheat (50%), corn, rice, oat, barley, rye, sorghum, and millet), minerals, natural flavor and vitamins

### Experimental Procedure and Measurements

The study uses a between-subjects experimental design and was carried out from October 2018 to January 2019. Parents were responsible for conducting the experiment and reporting on their children’s reactions and food intake. In-home studies present two advantages: they can be performed with few constraints on the participants, and children are in their usual environment with their usual feeder (55). Extant research has evidenced that parents are well aware of their infant’s responses toward foods and therefore are likely to be more sensitive to subtle differences in their reactions (15, 56–60). Parents were randomly allocated to one of the two groups: high-sugar refined (*n* = 82) and low-sugar whole grain cereals (*n* = 83).

**FIGURE 1:**
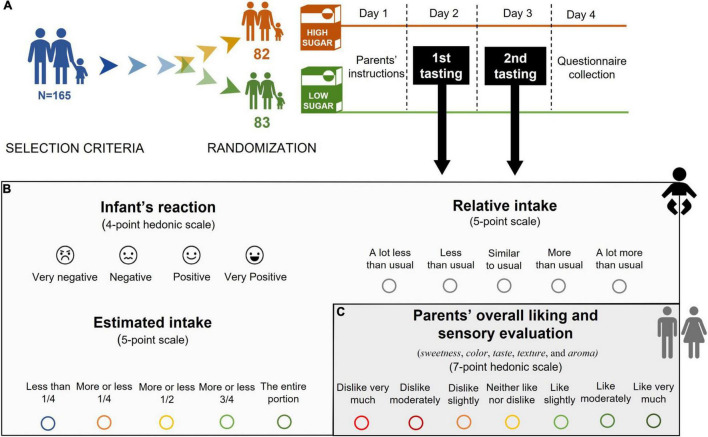
**(A)** Experimental design **(B)** Measurement of infant’s overall acceptability, and **(C)** parents’ overall liking and sensory evaluation.

The experiment was carried out during two consecutive days to have a more valid assessment of the reactions (as compared to a 1-day study). To be qualified for the study, infants had to be having commercial cereals at least 2 weeks before the experiment started. Prior to testing, to ensure accurate recording, parents were visited on day 1 by a research assistant who explained to them how to conduct the experiment, gave them clear and detailed written instructions, the questionnaire, and the infant cereals samples. Following standard procedures parents were given the following instructions: (1) to not feed their toddler with beverages or solid foods for 1 h before the testing so as not to influence their hunger status (58, 61), (2) to prepare cereals by mixing them with the infant formula milk that they had been using before the experiment took place, namely either follow-on formula (6–12 months) or growing-up milk (more than 12 months), which have very similar nutritional composition and properties. No further instructions were given in this regard as the aim was that infants were fed the very same way as they had been fed before the study took place so as not to bias the results (57), (3) to feed their infants in the habitual place, at a normal pace until the infant refused the spoon or bottle three consecutive times, (4) to fill out the evaluation of their infant’s reaction before conducting their own rating to avoid any possible bias and (5) to test and evaluate the cereals themselves only after infants have had theirs, in order ensure no interference with their infant’s reactions due to non-habitual parent behavior (product testing) during the feeding period. Parents’ reactions were included in our study because they play a major role in infant feeding practices and food brand/product selection (62, 63). Parents were also told to carry out the test the same time of days 1 and 2. No significant differences were found between the two groups (*F* = 4.21, *p* = 0.12) in terms of the moment of the day when the test took place (54.8% morning, 3.7% afternoon and 41.5% night in the high-sugar group vs. 43.4% morning, 10.8% afternoon and 45.8% night in the low-sugar group). On day 4, research assistants picked up questionnaires at the parents’ house. An overview of the study protocol is shown in Figure 1A.

#### Infants’ Overall Acceptability

Overall acceptability of cereals by infants was assessed through three different, yet related measures: the infant’s reaction toward the cereals (as perceived by parents), the estimated intake, and the relative intake of cereals compared to usual intake (see Figure 1B).

The infant’s reaction was measured by means of a 4-point hedonic scale (15, 32, 57, 58). The scale ranges from: “1 = very negative” if the infant spitted out the food, frowned, pushed the spoon away or stopped eating; “2 = negative” if the infant ate a couple of spoonfuls, grimaced and stopped eating; “3 = positive” if the infant ate some of the food without a specific reaction; “4 = very positive” if the infant accepted the first spoonful immediately and displayed signs of content, such as a relaxed face or a smile. All scores on the scale were accompanied with a corresponding smiley-face to guide the parents (Figure 1B).

The ingested amount (estimated intake) and the relative intake compared to usual infant cereal intake were measured *via* a 5-point scale with scores ranging from: “1 = less than 1/4” to “5 = the entire portion” and “1 = a lot less than usual” to 5 = a lot more than usual” (55) (Figure 1B).

#### Parents’ Rating of Overall Liking and Evaluation of Sensory Attributes

As depicted in Figure 1C, overall liking was measured using a one-item 7-point hedonic scale ranging from “1 = dislike very much” to “7 = like very much” (15, 57). Parents were also asked to evaluate key sensory attributes: sweetness, color, taste, texture, and aroma on the same 7-point hedonic scale (64).

#### General and Cereal Feeding Practices

Parents were asked for general feeding practices (e.g., age of first introduction of food, first food introduced) and cereal feeding practices (e.g., mode of consuming infant cereals, frequency of cereals intake, weight (g) of cereals prepared in one serving, brand of cereals used before the study). The wording of questions, sequence and response options were based on our previous work (65).

### Data Analysis

Differences between the feeding groups (high-sugar and low-sugar cereals) were tested with Student’s *t*-tests and Pearson chi-square tests. All results with a significance level of *p* < 0.05 were considered statistically significant. SPSS Version 26.0 (IBM, SPSS Inc., Armonk, NY, United States) software was used for statistical analyses.

## Results

### Participant Characteristics and Cereal Feeding Practices

The characteristics of the 165 parent-infant dyads are presented in Table 3. There were no significant differences between the two groups in terms of demographic characteristics.

**TABLE 3:** Demographic characteristics of infants and parents per group.

Variable	High-sugar cereals (*n* = 82)	Low-sugar cereals (*n* = 83)	*p*-value
**Infants**			
*Female (%)*	44	52	0.345
Age at inclusion (months) (mean ± SD)	11.0 ± 4.5	10.9 ± 4.8	0.830
*6–11 months (%)*	68	68	
*12–24 months (%)*	32	32	
**Parents**			
Age (years) (mean ± SD)	34.8 ± 5.1	35.3 ± 4.8	0.500
*20–30 years (%)*	22	16	
*31–40 years (%)*	65	69	
> *41 years (%)*	13	16	
Number of children (mean ± SD)	1.6 ± 0.8	1.7 ± 0.8	0.294
*1 (%)*	54	42	
*2 (%)*	32	44	
≥*3 (%)*	14	14	

**FIGURE 2:**
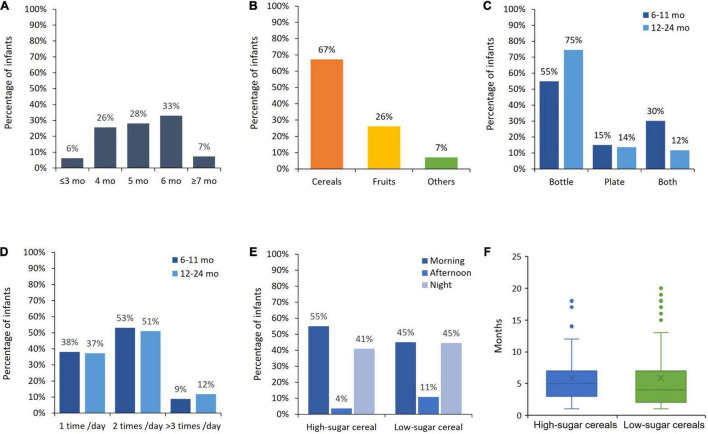
General and cereal feeding practices. **(A)** Timing (age) of introduction solids. **(B)** Types of first introduced solids. **(C)** Mode of infant cereal consumption. **(D)** Frequency of infant cereal consumption per day. **(E)** Moment of infant cereal consumption. **(F)** Duration in months of infant cereal consumption at inclusion.

No significant differences were found between the two groups in terms of general and cereal feeding practices, which further warrants the comparison between groups. As shown in Figure 2A, solids were mostly introduced between 4 and 6 months. Cereals (67%) were the first solid food introduced followed by fruits (26%) (Figure 2B). The mean age of introduction of cereals was 5.1 (± 1.2) months. The most frequent mode of preparation of cereals was the bottle (Figure 2C). Most of the infants (95%) consumed cereals daily (Figure 2D), mostly in the morning and/or at night (Figure 2E).

On average, the infants had been fed cereals 5.8 (± 4.2) months and 5.9 (± 4.9) months (high-sugar and low-sugar groups, respectively) before starting the experiment (Figure 2F). Importantly, these mean values were not significantly different (*F* = 1.54, *p* = 0.21) which further warrants comparing both groups. Infants had been given cereals with a similar sugar level which ranged from 15 to 25% in both groups. This implies that infants were used to having sweet cereals at inclusion.

### Infants’ Overall Acceptability

As shown in Table 4, there were no significant differences between the two groups in any of the three variables used to measure infants’ overall acceptability (infant’s reaction, estimated intake and relative intake) on either day of the experiment (days 1 and 2).

**TABLE 4:** Infants’ overall acceptability (differences by group on mean values).

		High-sugar cereals	Low-sugar cereals	*p*-value
Infants’ reaction*	Day 1	3.21 ± 0.72	3.31 ± 0.64	0.318
	Day 2	3.24 ± 0.68	3.34 ± 0.69	0.380
	*p*-value	0.320	0.483	
Estimated intake**	Day 1	4.35 ± 1.07	4.39 ± 1.11	0.851
	Day 2	4.38 ± 1.00	4.36 ± 1.17	0.922
	*p*-value	0.726	0.709	
Relative intake***	Day 1	2.87 ± 0.52	2.96 ± 0.53	0.230
	Day 2	2.93 ± 0.54	2.95 ± 0.47	0.751
	*p*-value	0.167	0.783	

*4-point hedonic scale from “1 = very negative” to “4 = very positive”; **5-point scale from “1 = less than 1/4” to “5 = the entire portion”; ***5-point scale: “1 = a lot less than usual” to “5 = a lot more than usual”.

**FIGURE 3:**
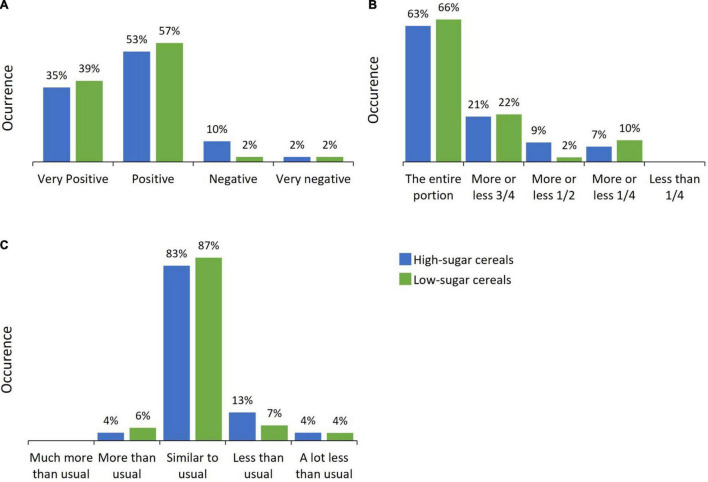
Infants’ overall acceptability (differences by group in percentages). **(A)** Infant’s reaction. **(B)** Estimated intake. **(C)** Relative intake.

In addition, as illustrated in Figure 3A, the frequency of positive and very positive reactions reported by parents was high in both groups (88 and 96% in the high-sugar and low-sugar group, respectively). Similarly, estimated intake was high (more than 60%) in both groups (Figure 3B) and relative intake was mostly similar to usual (before the start of the experiment) in both groups (83 and 87%, respectively as shown in Figure 3C). Overall, these results highlight the very good acceptability of low-sugar cereals by infants in our study.

**FIGURE 4:**
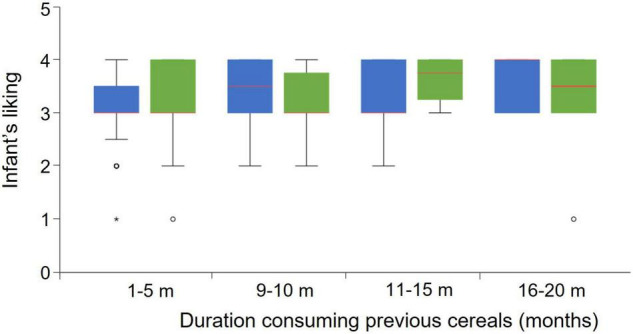
Influence of duration consuming previous cereals on infant’s reaction. Dots are mild outliers (Q1-1.5*IQR). The asterisk is an extreme outlier (Q1-3*IQR).

To further substantiate our results, we examined the extent to which the number of months that infants had been exposed to sweet cereals (15–25% sugar) before the beginning of the study could have an influence on infants’ reactions to high- and low-sugar cereals in our experiment. We did not find any significant differences in infants’ reactions depending on the duration they had been consuming cereals before the experiment took place (Figure 4).

### Parents’ Rating of Overall Liking and Evaluation of Sensory Attributes

Consistent to findings reported above on infants’ overall acceptability, parents’ overall liking and sensory evaluation were positive and similar in both groups in all attributes considered. More specifically, as reported in Table 5, differences between the two groups were not significant.

**TABLE 5:** Parents’ rating of overall liking and evaluation of sensory attributes (differences by group on mean values).

Attributes*	High-sugar cereals	Low-sugar cereals	*p*-value
Overall liking	5.35 ± 1.33	5.40 ± 1.14	0.796
Color	5.52 ± 1.19	5.75 ± 1.09	0.188
Aroma	5.76 ± 1.34	5.92 ± 1.14	0.429
Taste	5.48 ± 1.44	5.60 ± 1.16	0.535
Sweetness	5.48 ± 1.43	5.36 ± 1.29	0.552
Texture	5.85 ± 1.23	5.79 ± 1.38	0.774

*7-point hedonic scale: “1 = dislike very much” to “7 = like very much”.

### Differences in Cereals and Sugar Intake per Group

As shown in Table 6, the mean quantity of cereals intake per day was significantly larger in the high-sugar group (27.42 g) as compared to the low-sugar group (19.22 g). This larger intake of cereals, along with a much higher content of sugar, implies that sugar intake per day is more than 30 times larger in the high-sugar group (5.77 g), in comparison to the low-sugar group (0.19 g). Furthermore, the consumption of free sugars would be 3.40% of total daily energy intake in the high-sugar group, and only 0.12% in the low-sugar one. If we extrapolate these figures to annual consumption, the estimated yearly intake of sugar would be around 2 kg in the high-sugar group as compared to 69 g in the low-sugar group.

**TABLE 6:** Sugar intake from cereals (differences by group).

	High-sugar cereals	Low-sugar cereals	*p*-value
Cereal intake (g/day)	27.42 ± 15.41	19.22 ± 13.94	0.001
Sugar intake from cereals (g/day)	5.77 ± 3.23	0.19 ± 0.14	0.001
% energy from sugar in cereals*	3.40 ± 1.93	0.12 ± 0.08	0.001
Estimated yearly intake of sugar (g)**	2093.77 ± 1179.7	69.26 ± 51.71	0.001

*This value was calculated by (1) calculating the sugar intake from cereals in g/day: [mean cereal intake g/day] × [sugar content high- OR low-sugar cereal]/100, (2) converting sugar intake from cereals in g/day to kcal/day by multiplying the value by (4), and (3) calculating the contribution of energy from sugar in kcal/day relative to recommended total energy intake in line with age and gender (77): [sugar intake from cereals kcal/day] × 100/[mean recommended total energy intake kcal/day].

** [cereal intake g/day] × [number of days consuming cereals in a week] × 52 weeks.

## Discussion

This study examined infants and their parents’ acceptability of a high-sugar refined cereal vs. low-sugar whole grain cereal. We found no significant differences between the two groups in terms of infants’ overall acceptability (infant’s reaction, estimated and relative cereals intake). Importantly, infants’ reactions to high- and low-sugar cereals were not influenced by the time that infants had been consuming sweet cereals (15–25% sugar) before the experiment took place. In addition, parent’s overall liking and sensory evaluation (sweetness, color, taste, texture, and aroma) were not significantly different between groups. Overall, our findings suggest that it is feasible to reduce sugar content in infant cereals (and add whole grain) without sacrificing its sensory acceptability by infants (and their parents).

### Theoretical and Practical Implications

The reduction of sugar levels can be extremely challenging for food manufacturers because of the expected changes in food sensory characteristics (7). Only recently, as described in the introduction, sensory and consumer scientists have paid attention to testing consumers’ reactions to sugar-reduced food products. Most of these studies have been conducted with adults and to a minor extent with children, who tried the reformulated (sugar-reduced) products only once. On the contrary, we focused on infants who tested the reformulated products more than once during 2 days, thus improving the reliability of the results. Our findings concur with those studies which evidenced that sugar reductions are feasible (13, 14, 16, 17). However, as infant cereals were prepared with infant formula, which contains about 7 g of lactose/100 ml, we could hypothesize that their sweet taste might improve the acceptability of this large sugar reduction (95.2%) (15, 57) in comparison with other studies where smaller reduction failed to do so (11, 20, 26, 28, 29). This implies that the product category to be reformulated represents a key factor which significantly affects the extent to which sugar levels can be reduced and still be accepted by consumers. For example, recent findings from Klerks et al. reveal that a reduction of sugar content up to 30% in yogurt pouches is sensory accepted by toddlers (1–4 years old) and their parents (17). Furthermore, our results build and significantly add to Sanchez-Siles et al.’s (15) study in many ways. First, we test a much larger sugar reduction (95.2 vs. 50%). Second, we evaluate a wider spectrum of sensory reactions both in infants and their parents. Third, we use a larger sample of infants (165 vs. 46) from several cities (5 vs. 1). Fourth, and most importantly, infants in our study were already used to having sweet cereals (15–25% sugar), whereas infants in Sanchez-Siles et al.’s (15) had never tried cereals before. Notably, our findings along with previous results from Sanchez-Siles et al. (15) and Haro-Vicente et al. (57) demonstrate that the addition of whole grain does not affect infant cereal’s acceptability (15, 57).

Overall, the insights offered in this study are particularly relevant as they indicate that infants (in these early stages of their life), even used to having sweet cereals, can accept cereals that are highly reduced in sugar. This provides further evidence to previous studies suggesting that even though infants are born with an innate preference for sweet taste, such preferences are likely to be modified during the complementary feeding period (34, 66). This is one of the reasons why several international health agencies and pediatric associations recommend stricter regulations regarding sugar intake in infants and children. For example, in 2015 the WHO recommended that the consumption of free or added sugars should not exceed 5% of total daily energy intake for children below 2 years, while the ESPGHAN Committee on Nutrition recommended that sugar should not be added to complementary foods (3). Similarly, the American Heart Association [AHA] (4, 46) and the new Dietary Guidelines for Americans (47) recommended to avoid the consumption of any added sugar in children younger than 2 years old, and a Policy brief from the WHO Regional Office for Europe (5) has recently called for a complete prohibition of added, free sugars and sweeteners (including syrups, honey, fruit juice, fruit juice concentrates, and non-sugar sweeteners) in all commercial complementary foods.

Surprisingly, most of these organizations and agencies focus the guidelines on “free sugars,” but none of them have considered the sugars produced during the hydrolyzation of cereals. The level and type of sugar produced depend on the degree of hydrolysis (time and temperature) and the type of enzyme used. The use of gluco-amylase improves the sweetness (due to the production of glucose), while the use of alpha-amylase produces dextrins and maltose (less sweet than glucose and sucrose). Depending on the degree of hydrolysis the sugar level in cereals can reach up to 30% of sugar (10). This is particularly disturbing because sugar produced while hydrolyzing cereals is not specifically mentioned or defined in any current legislation around the world. As a result, some manufacturers claim that they are producing cereals “with non-added sugar,” while they may have a very high content of produced sugar as a result of the hydrolysis process. Therefore, we strongly encourage policymakers to consider sugars produced in the hydrolysis as free sugars.

We found cereals to be the first solid foods introduced (in 67% of the participants in our study), in line with previous studies in Spain (65) and other countries such as United Kingdom, Ireland and Canada (67–69). Also, our results showed that the frequency of intake of infant cereals was high (95% of infants consumed cereals daily and around 50% were fed with cereals two times per day). Thus, cereals may represent the first time infants try solid foods with added and/or produced sugar. Importantly, daily consumption of cereals (and sugar) was significantly higher in the high-sugar group (hydrolyzed cereal) as compared to the low-sugar group (non-hydrolyzed cereal). The lower intake observed is directly related to the presence or not of hydrolysis. That is, when starch is hydrolyzed, the viscosity is reduced, and consequently more grams of cereals have to be added to obtain the same texture properties (mainly viscosity) of the cereals without hydrolysis. Therefore, a reduction of 95.2% of sugar as tested in our study would imply that infants would be reducing their sugar intake with more than 2 kilos yearly (see Table 6). This estimated reduction would be directly in line with the development and promotion of sustainable healthy diets (70–72), and would make it much easier to comply with the WHO recommendation of sugar consumption not exceeding 5% of total daily energy intake. Furthermore, the elimination of hydrolysis would represent a significant reduction in food processing, leading to less-processed, more natural products consistent with the latest food international consumers’ trends (73, 74).

### Limitations and Future Research Opportunities

While our study offers important implications, we recognize some limitations which can lead to interesting future research directions. First, unlike prior research (15), we conducted our experiment with infants and parents located in five different Spanish cities. Still, our findings need to be validated in other countries with special emphasis on contexts where infant cereals are characterized by a high sweetness intensity (e.g., Middle East countries). Second, following extant research (18, 28, 29), our study relied on a between-subject design where each group tested only one product (either non-reformulated or reformulated cereals). Importantly, both groups can be considered as equivalent (75), as there were no significant differences in terms of the relevant characteristics for the topic under research (e.g., number of months having cereals at inclusion, age, gender of the subjects). Future research could try to replicate our findings through a repeated measures design where the same group of subjects (infants and their parents) test both products. Third, infants (and their parents) tested the cereals several times over two consecutive days and results did not differ between days 1 and 2. Still, participants could try the cereals for a longer period in future studies. Finally, our analyses were focused on complementary infant cereals. Future research is needed to examine infants’ reactions to sugar reductions in other product categories such as snacks (some of them characterized by high levels of sugar) and where sugar function is not restricted to delivering a sweet taste, but also providing bulk and texture (76).

## Conclusion

Findings from our study indicate that it could be possible to significantly reduce sugar content (or even eliminate it) and add whole grain in infant cereals without influencing infants’ overall acceptability. This is extremely relevant, as prior research has shown that exposure to low-sugar foods in infancy may promote the acceptance of and eventually a preference for those foods across the lifespan. The current study represents a promising case for the infant food industry to adhere to current healthy and sustainable demands leading to important benefits in infants’ health, without compromising competitiveness in the market.

## Data Availability Statement

The raw data supporting the conclusions of this article will be made available without undue reservation, upon request to the corresponding author.

## Ethics Statement

The studies involving human participants were reviewed and approved by the Research Ethical Committee of the University of Murcia (code: CEI 2116/2018). Written informed consent to participate in this study was provided by the participants’ legal guardian/next of kin.

## Author Contributions

LS-S: contributes to the conceptualization, methodology, supervision, writing—original draft, writing—review and editing. SR: contributes to the conceptualization, writing—original draft, writing—review and editing. JH-V: contributes to the data curation, formal analysis, investigation, methodology, writing—review and editing. MB: contributes to the conceptualization, data curation, formal analysis, methodology, and writing—review and editing. MK: contributes to the writing—original draft and writing—review and editing. GR and ÁG: contributes to the conceptualization and writing—review and editing. All authors read and approved the final manuscript and agreed to be accountable for the content of the work.

## Conflict of Interest

LS-S, JH-V, MB, and MK were members of the Research and Nutrition of Hero Group, a Swiss international food manufacturer; ÁG received honoraria-payments for specific speeches and seminar presentations from Hero, Nestlé; Lactalis and others baby food companies. The remaining authors declare that the research was conducted in the absence of any commercial or financial relationships that could be construed as a potential conflict of interest. The authors declare that this study received funding from Hero ÁG. The funder was not involved in the study design, collection, analysis, interpretation of data, the writing of this article or the decision to submit it for publication.

## Publisher’s Note

All claims expressed in this article are solely those of the authors and do not necessarily represent those of their affiliated organizations, or those of the publisher, the editors and the reviewers. Any product that may be evaluated in this article, or claim that may be made by its manufacturer, is not guaranteed or endorsed by the publisher.
